# Common Causes of Anaphylaxis in Children: The First Report of Anaphylaxis Registry in Iran

**DOI:** 10.1097/WOX.0b013e3181c82128

**Published:** 2010-01-15

**Authors:** Saeideh Barzegar, Akramian Rosita, Zahra Pourpak, Mohammad Hassan Bemanian, Raheleh Shokouhi, Mahboubeh Mansouri, Taher Cheraghi, Zahra Chavoshzadeh, Iraj Mohammadzadeh, Mohammadreza Fazlollahi, Bahram Mirsaeedghazi, Mohammad Nabavi, Masoud Movahedi, Mohammad Gharagozlo, Fatemeh Farahmand, Mostafa Moin

**Affiliations:** 1 The Immunology, Asthma and Allergy Research Institute, Tehran University of Medical Sciences, Tehran, Iran; Department of Immunology and Allergy, Pediatrics Center of Excellence, Children's Medical Center, Tehran University of Medical Sciences, Tehran, Iran; Pediatric Department, Shahid Sadoughi Hospital, Yazd University of Medical Sciences, Yazd, Iran; Immunology and Allergy Department, Mofid Children Hospital, Shahid beheshti University of Medical Sciences, Tehran, Iran; Children Research Institute, Babol University of Medical Sciences, Babol, Iran; Pediatric Department, Bahrami Hospital, Tehran University of Medical Sciences, Tehran, Iran; Pediatric Department, Amirolmomenin Hospital, Semnan University of Medical Sciences, Semnan, Iran; Gastroenterology Department, Pediatrics Center of Excellence, Children's Medical Center, Tehran University of Medical Sciences, Tehran, Iran; 2 Supported by the Immunology, Asthma and Allergy Research Institute (IAARI) in Tehran, Iran; 3 Children's Medical Center, Immunology, Asthma and Allergy Research Institute, Keshavarz blvd, Dr Gharib ave, Tehran, Iran

**Keywords:** anaphylaxis, common causes, children

## Abstract

**Background:**

Anaphylaxis is an acute, systemic, and potentially fatal allergic reaction. Many things can cause anaphylaxis potentially but some agents are more common like some foods (milk, egg, soy, wheat, peanut, tree nut, shellfish, and fish), insect stings, medications, latex, and food-dependent exercise-induced anaphylaxis. The goal of this study is to show the common causes of anaphylaxis among the children with anaphylaxis history who were referred to the Immunology, Asthma and Allergy Research Institute (IAARI) during a 4-year period (2005-2009).

**Methods and Materials:**

During those 4 years, we registered all children (<14 years old) with a history of anaphylactic reaction. To prove the cause of anaphylaxis, we performed skin prick tests with suspected agents according to their history and measured specific IgE against them by the ImmunoCAP test. Recognition of common allergens was based on having a positive history for 1 allergen and positive skin prick test or specific IgE for that at the same time, or having positive results from both tests when the allergen was unclear. Idiopathic anaphylaxis was a reaction when any known allergen and positive tests were not obtained.

**Results:**

One hundred ninety-three nonfatal anaphylactic attacks among 63 children were recognized. In total, the most current cause of anaphylaxis in children was food (89.7%). Milk (49.3%) and wheat (26.1%) were the most common. Other foods were egg (8.7%), nuts (2.8%), and spices (2.8%). Six children (8.7%) were sensitive to multiple food allergens like milk, egg, and wheat. Five (7.1%) of 63 patients had anaphylactic attack because of stinging. Wasp was the trigger in 3 (4.3%) of them and honeybee was the cause in 1 (1.4%). The last one was because of unknown hymenoptera. There were 2 idiopathic cases of all 63 patients.

**Conclusions:**

Food allergens, especially milk and wheat, are the most common cause of anaphylaxis in children. Because anaphylaxis can be fatal, it is advisable to recognize its causes in different communities to prevent recurrent attacks.

## 

Anaphylaxis is an acute, systemic, and potentially fatal allergic reaction. It happens when an allergen interacts with specific IgE found on tissue mast cells and blood eosinophils. Then histamines and other mediators are released and symptoms like flushing, urticaria, angioedema, stridor, vomiting, dizziness, hypotension, shock, and even death can happen. These problems occur within seconds or minutes of antigen exposure [1]. Anything can cause anaphylaxis potentially but some agents are more common like foods (milk, egg, soy, wheat, peanut, tree nut, shellfish, and fish), insect stings (honeybees, bumblebees, sweat bees, yellow jackets, hornets, wasps, and ants), medications, and latex. There is also food-dependent exercise-induced anaphylaxis in some cases [2]. When there is no identifiable agent or event to cause anaphylaxis, it is called idiopathic anaphylaxis [3].

It seems that the causes of anaphylaxis are different between children and adults. Drug-induced--especially NSAIDs and antibiotics--anaphylaxis, idiopathic anaphylaxis, and food-dependent exercise-induced anaphylaxis are more frequent in adults whereas foods are the leading single cause of anaphylaxis in children [1,4].

The purpose of this study is to understand the common causes of anaphylaxis in children who were referred to the Immunology, Asthma and Allergy Research Institute (IAARI) in Iran during a 4-year period from January 2005 to January 2009. A quicker and correct diagnosis of anaphylaxis in the children of our area would be quite helpful and therefore allow us to help them prevent and control the anaphylaxis events.

## Methods and materials

IAARI is an Iranian research institute that is a main referral center for patients with allergies. The definition of "child" or "children," with respect to our study, is 14-years old and younger [5]; hence, all children younger than 14 years old referred to IAARI during the stated 4-year period (2005-2009) with a positive history of anaphylactic reaction and who had inclusion criteria and did not have exclusion criteria were included in this study. The inclusion and exclusion criteria are mentioned next.

### Inclusion criteria

• The patients should have at least 2 significant signs or symptoms of anaphylaxis after contact with suspected agent.

• The signs and symptoms are mucocutaneous symptoms (urticaria, angioedema, flushing, and pruritus), respiratory symptoms (cough, bronchospasm, hoarseness, glossal, pharyngeal, or laryngeal edema, wheezing, and dyspnea), cardiovascular symptoms (tachycardia, hypotension, and shock), gastrointestinal symptoms (nausea, vomiting, diarrhea, and abdominal cramps), and neurologic symptoms (dizziness, drowsiness, and decrease in level of consciousness) [1,6].

• The onset of signs and symptoms should be acute.

### Exclusion criteria

• The signs and symptoms are due to some other illnesses (differential diagnosis of anaphylaxis). For example,

• hypotension due to septic shock, cardiogenic shock, hypovolemic shock, and vasovagal reflex

• histamine syndromes like systemic mastocytosis and basophyilic leukemia

• flushing of carcinoid tumor

• Redman syndrome from vancomycin

• panic attack, stroke, familial angioedema, pheochromocytoma, and hydatic cyst (differential diagnosis of anaphylaxis) [7,8]

At the first visit in IAARI for each child a questionnaire was filled out by a general practitioner to provide complete information including the patient's demographic data, personal history of other kinds of allergic diseases like rhinitis, atopic dermatitis, and asthma, trigger of anaphylaxis, number of anaphylactic attacks, and clearly their signs and symptoms related to anaphylaxis. The obtained information from their histories and tests were analyzed by the spss program.

After taking informed consent from parents, we performed a set of laboratory tests that included the following:

1. *Measurement of specific IgE against suspected allergen(s) based on the patient's history by the ImmunoCAP method: *This test was done by laboratory substances from Phadia AB, Uppsala, Sweden. The results were read by Immuno-CAP 100. The responses were graded between 0 and +6 on the basis of the kit's procedure [9]. To do this test, we took a 2-to 3-mL clot and detached the serum for measuring specific IgE. The serum was reserved at −20°C. The CAP test was performed for all the patients.

2. *SPT (skin prick test): *This test was done on the volar side of the forearm. In this test, on the basis of the patient's history, we put a drop of suspected allergen(s) on the volar side of the patient's forearm and then with a lancet via a prick the allergen penetrates. As positive and negative controls, 1 mg/ml of histamine and normal saline were used. If positive control was positive for at least 5 mm and negative control was negative, the skin test would be performed. The skin test is read after 15 minutes: a wheal diameter greater than 3 mm is positive and less than 3 mm is negative. Skin test agents were standard extract from Allergopharma Co., Reinbeck, Germany. This test was done under observation of an immunologist with resuscitation conditions [10]. SPT would be performed 6 weeks after anaphylactic reaction for those who had negative specific IgE.

In the cases of those children from Iran who did not have a clear history of anaphylaxis trigger, CAP and SPT were done for some current food allergens, for example, milk, egg, soy, and wheat [11].

### Explanation of tests and discovery of allergens

1. After the patient's history was taken and specific IgE or skin prick tests were performed, if the history and one of the CAP tests for specific IgE or SPT was positive for an allergen, that allergen would be known as the cause of anaphylaxis.

2. If the patient did not know the exact trigger of reaction but both the specific IgE test and the skin prick test were positive for one probable allergen based on the patient's food diary, that allergen was recognized as the cause of anaphylaxis [12,13].

3. If one clear allergen, skin prick test, or specific IgE test did not specify the cause of anaphylaxis, but a clinical feature introduces an anaphylaxis attack, we named the cause "idiopathic." [14]

## Results

The number of children with at least one anaphylactic attack who were referred to IAARI from January 2005 to January 2009 was 63.

The minimum age was 3 months and the maximum age was 156 months. The mean age was 39.63 months.

There were 46 (73%) boys and 17 (27%) girls in this group of children.

The percentage of patients that had a positive history of other kinds of allergic disease was 36.5%.

With regard to the explanation of the tests and discovery of the allergens in this method:

• Sixty children had positive anaphylaxis history because of at least 1 clear allergen and positive CAP test for that allergen.

• Twenty-four children had positive anaphylaxis history because of at least 1 obvious allergen and positive SPT for that allergen.

• Five children had both positive CAP test and SPT without a clear trigger for anaphylaxis.

• There were a total of 2 idiopathic cases.

**Figure 1:**
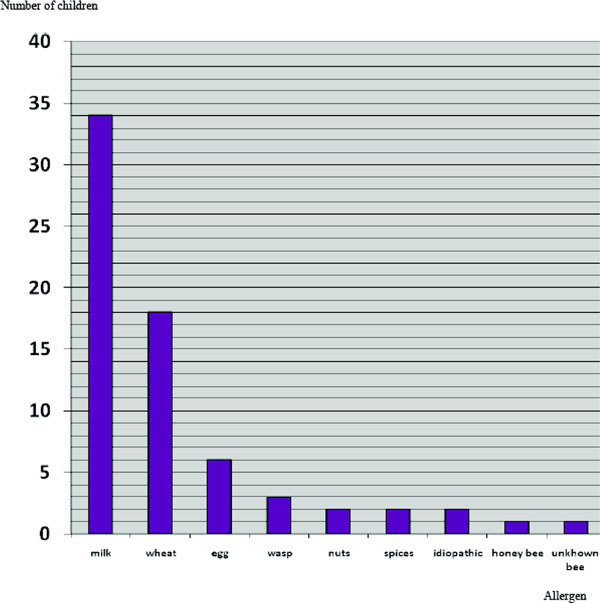
**Causes of anaphylaxis in children**.

In total, the most current cause of anaphylaxis in children was food (89.7%). After food, stinging of hymenoptera was the second most common cause of anaphylaxis. The details are shown in Figure 1.

Six children (8.7%) had a history of anaphylaxis because of multiple food allergens like milk, egg, and wheat.

The number of anaphylactic attacks related to each cause are presented in detail in Table 1.

**Table 1:** Prevalence of Allergens and Number of Anaphylactic Attacks Related to Them in 63 Children

Allergen	No. of Children	Percentage	No. of Anaphylactic Attacks	Percentage
Food				
Milk	34	49.3	102	52.85
Wheat	18	26.1	59	30.56
Egg	6	8.7	10	5.2
Nuts	2	2.8	5	2.59
Spices	2	2.8	4	2.08
Hymenoptera				
Wasp	3	4.3	3	1.55
Honey bee	1	1.4	1	0.52
Unknown bee	1	1.4	1	0.52
Idiopathic	2	2.9	8	4.15
Total	69	100	193	100

One hundred ninety-three nonfatal anaphylactic attacks among 63 children were recognized; 46 (74.19%) of the children reported more than 1 anaphylactic reaction related to an obvious allergen (the details are shown in Table 2). However, the higher number of anaphylactic attacks in 1 patient were because of wheat (2 cases had 7 anaphylactic attacks and 1 case had 10 attacks). Those children that were sensitive to multiple food allergens had at least 2 attacks.

**Table 2:** Number of Children Related to the Number of Anaphylactic Attacks

No. of attack(s)	No. of Children	Total Attacks
1	15	15
2	17	34
3	16	48
4	4	16
5	6	30
6	3	18
7	2	14
8	1	8
9	0	0
10	1	10
Total	63	193

Mucocutaneous signs and symptoms were the most common of all anaphylactic symptoms (92/2%). Urticaria was seen in 54/9%, flushing in 43/1%, angioedema in 41/2%, and pruritus in 56/9%. Respiratory problems were the second most common (76/5%). We defined respiratory symptoms as cough (27/5%), stridor (3/9%), sneezing (17/6%), difficultly speaking (9/8%), wheezing (56/9%), and dyspnea (70/6%).

Gastrointestinal, cardiovascular, and neurologic manifestations were less frequently present.

We encountered no fatalities attributable to anaphylaxis.

We did specific IgE tests (ImmunoCAP) for all children and 60 (95.2%) of the tests were positive.

We did skin pricks for 26 children, and in 24 of them, the results were positive.

## Discussion

Each country has its own rule of diet so the common causes of anaphylaxis can be different in each country, and so it is necessary to know them because anaphylaxis is acute and potentially fatal [15].

Documented studies about the epidemiology of anaphylaxis causes in Asian areas are limited [16].

There are not enough studies about the epidemiology and prevalence of allergens that cause anaphylaxis in Iran. In 1 study of wheat allergy in children in Iran, clinical symptoms of 54% of children sensitive to wheat appeared as anaphylaxis [17].

There was also a study in Iran by Pourpak et al that reports, in detail, wheat anaphylaxis in 19 children. It indicates that wheat-induced anaphylaxis is very severe and not preventable[18].

In our study, like other studies, anaphylaxis was more common in boys [19].

Foods and significantly milk were the most current causes of anaphylaxis in this study. It seems similar to previous studies but there were also some differences.

One study by Braganza in 2006 indicated that food allergy was the most common cause of anaphylaxis in children in the emergency ward [20].

It is estimated that in the United States 30,000 anaphylactic reactions and 150 to 200 deaths due to food anaphylaxis occur yearly [21].

In Novembre's study about anaphylaxis in children, the most common foods that cause anaphylaxis were fish, cow's milk, nuts, egg, fruit, cereals, vegetables, and goat's milk [19].

In England, a study showed that fatal reactions in children are caused by cow's milk and those in teenagers are cause by peanuts [22].

As reported in Lane's study, among foods, peanut is the most common cause of anaphylaxis in children in the United States, in Australia egg, peanut, and dairy products, in Italy shellfish and dairy products, and southeast Asian countries shellfish [4].

Howeve, in our study cow's milk and then wheat are the top causes of anaphylaxis in children in Iran whereas the prevalence of anaphylaxis induced by peanut and fish or shellfish is very low. This can be due to the differences in nourishment in Iran where eating seafood is not routine.

The high number of anaphylactic attacks, 193, among 63 children, on average 3.06 attacks for each child, can show a parent's unawareness of the cause and prophylaxis of anaphylaxis.

We had only 2 cases that had idiopathic anaphylaxis and it is compatible with other studies. Idiopathic anaphylaxis is more common in adults and rarely occurs in children [1].

Most of the cases had repeated anaphylactic attacks at different levels of severity. Possibly, it was because of limited knowledge about anaphylaxis and its causes and some believe that people think it had been just an accidental reaction and would not occur again or if they use that agent more they will become insensitive and resistant to that allergen.

There were also some limitations for our study. Our institute is a research and referral center for allergic patients, so our sample size is much less than the true number of children with anaphylaxis in that period; also we registered the children's symptoms by taking a history from their parents or in their letter of introduction.

## Conclusions

In our study, like many studies in all the world, food allergy was the most common and important cause of anaphylaxis in children and between the foods milk and then wheat and egg were more common. Wheat and especially milk are 2 essential foods for children so parents and physicians should be aware of the possibility of anaphylaxis symptoms so that the necessary treatment can be administered quickly and steps can be taken to prevent recurrences.
